# FAM60A promotes osteosarcoma development and progression

**DOI:** 10.1002/cam4.6343

**Published:** 2023-07-12

**Authors:** Yu Sun, Yu‐Nan Man, Jin‐hui Cheng, Jing‐tang Li, Ya‐yun Liu

**Affiliations:** ^1^ Division of Spinal Surgery The First Affiliated Hospital of Guangxi Medical University Nanning Guangxi Zhuang Autonomous Region P.R. China; ^2^ Jiangxi Provincial People's Hospital The First Affiliated Hospital of Nanchang Medical College Nanchang Jiangxi China

**Keywords:** clinical significance, family of homology 60A, hub genes, osteosarcoma

## Abstract

**Background:**

Osteosarcoma (OS) is a highly malignant primary bone tumor. Family of homology 60A (FAM60A) reportedly contributes to the malignant growth of some tumors.

**Methods:**

Herein we investigated the mRNA expression level of FAM60A by combining OS and non‐cancer samples from public databases. Immunohistochemistry was performed to determine protein expression levels of FAM60A in patients with OS. Further, RT‐qPCR and western blotting were conducted to evaluate FAM60A expression in various OS cell lines. CCK‐8 assay, colony formation assay, and flow cytometry were applied to determine the function of FAM60A. Finally, functional enrichment analysis was performed based on FAM60A co‐expressed genes.

**Results:**

FAM60A mRNA expression level was found to be significantly upregulated (standardized mean difference = 1.27, 95% CI [0.67–1.88]). Survival analyses suggested that higher expression of FAM60A was indicative of poor prognoses. Similarly, FAM60A protein expression level was also observed to be upregulated. Knocking down FAM60A expression inhibited OS cell proliferation, increased apoptosis, and blocked cells from entering the S phase. Besides, cell cycle was the most prominently enriched pathway, and BUB1, DTL, and EXO1 were identified as hub genes.

**Conclusions:**

FAM60A expression was found to be markedly upregulated in OS; furthermore, FAM60A was observed to promote OS cell proliferation, inhibit apoptosis, and participate in cell cycle regulation. Besides, FAM60A may interact with hub genes to participate in the progress of OS.

## 1 INTRODUCTION

Osteosarcoma (OS), a type of primary malignant bone tumor, often develops from mesenchymal cells. OS most commonly arises from the metaphyseal end of long bones, such as the distal femur, proximal tibia, and humerus. It is characterized by rapid cell proliferation and invasion, high risk of metastasis, and high morbidity in children as well as adolescents.^1^, ^2^, ^3^ At present, the conventional treatment for OS is neoadjuvant chemotherapy and surgery (amputation and limb‐salvage surgery) plus chemotherapy.^4^, ^5^ However, current treatment methods are still associated with dismal clinical outcomes; studies have reported that the survival rate of patients with OS has almost remained unchanged in the last two decades.^6^, ^7^ In recent years, clinical treatment strategies for malignant tumors have witnessed considerable improvement, and molecularly targeted therapy has emerged to be very effective.^8^ Accordingly, identifying new biological markers and molecular therapeutic targets can provide new insights and ideas for treating OS.

FAM60A (Family of homology 60A), located on human chromosome 12p11.21 and considered to be a subunit of the SIN3A/HDAC (histone deacetylase) complex, usually binds to this complex and actively participates in cell proliferation, differentiation, metastasis, and cell cycle regulation.^9^, ^10^ Streubel et al. reported that loss of the FAM60A pseudo‐phenotype in embryonic stem cells caused SIN3A inactivation, consequently reducing the proliferation rate of these cells and prolonging the G1 phase.^11^ Numerous studies have demonstrated a strong correlation between the progression of cancer and abnormal expression of FAM60A, as well as drug resistance. For instance, Dong et al. found that FAM60A expression was upregulated in esophageal cancer which was positively correlated with tumor size, lymph node metastasis, and TNM stage.^12^ Moreover, the upregulation of FAM60A expression has been linked to worse prognosis of patients with gastric cancer. Further, *Helicobacter pylori* infection upregulates FAM60A expression by targeting the PI3K/AKT pathway, enhancing the development of gastric cancer cells.^13^ FAM60A can evidently bind to the promoter of TGF‐β receptor type 1, playing a key role in the transcriptional repression of hepatocellular carcinoma; in addition, it has been reported that deletion or decreased expression of FAM60A promotes metastasis of hepatocellular carcinoma cells.^14^, ^15^, ^16^ According to some studies, FAM60A overexpression promotes cisplatin resistance in lung cancer cells, which involves upregulation of the expression of multidrug resistance gene 1.^17^, ^18^ In summary, FAM60A plays a role as an oncogenic gene in esophageal cancer and gastric cancer, and as an inhibitory gene in hepatocellular cancer. Further, it can also promote cisplatin resistance in lung cancer. These insights present a new direction for tumor research and novel approaches for the detection and treatment of cancer based on the diverse roles played by FAM60A in cancer progression.^19^ However, there is a lack of pertinent studies on the role of FAM60A in OS.

Therefore, herein our aim was to investigate the clinical significance and molecular functions of FAM60A in OS. We first determined the mRNA expression level of FAM60A by integrating OS public datasets, and the clinical significance and protein expression level of FAM60A in OS were explored by immunohistochemistry (IHC). The expression level of FAM60A in four OS cell types was then determined by real‐time quantitative PCR (RT‐qPCR) and western blotting (WB). Subsequently, to precisely elucidate the biological function of FAM60A in OS, various experiments were performed to assess cell proliferation, colony proliferation, cell apoptosis, and cell cycle.

## 2 MATERIALS AND METHODS

### 2.1 Integration of OS public datasets

Gene Expression Omnibus (GEO) and ArrayExpress were used to obtain FAM60A expression data in OS. The search terms included “(osteosarcoma or osteogenic sarcoma) and mRNA and (*Homo sapiens*).” The inclusion criteria were as follows: (1) studies comprising both an OS group and a non‐cancer group and (2) patients should not have received chemotherapy, radiotherapy, or any other treatment prior to tissue collection. The exclusion criteria were as follows: (1) studies assessing nonhuman samples or cell lines and (2) studies without any OS‐related FAM60A expression data. To determine the prognostic value of FAM60A in OS, we used the TARGET (https://ocg.cancer.gov/programs/target) public database to obtain OS mRNA expression and outcome data. Additionally, we obtained single‐cell datasets of OS from the TISCH2 database and investigated the expression of FAM60A in the OS microenvironment.^20^

### 2.2 Process for standardization of the OS datasets

The gff3 file downloaded from GENCODE (https://www.gencodegenes.org/human/) was used to annotate the datasets included in this study. We filtered the samples with an average expression level of 0 and performed log_2_ (x + 1) transformation for data that were not standardized in order to confirm that they approximately conformed to a normal distribution pattern. Finally, for RNA‐seq datasets that were only provided in the FPKM (fragments per kilobase of exon model per million mapped fragments) format, we converted FPKM values to TPM (transcripts per kilobase of exon model per million mapped reads) values with the following transformation formula: $\mathrm{TP}M_{i}=\left(\frac{\mathrm{FPK}M_{i}}{\sum_{j}\mathrm{FPK}M_{j}}\right)\cdot 10^{6}$.

### 2.3 Immunohistochemistry (IHC)

Two tissue microarrays, L1024901 and LN020Bn01, were obtained from Guanghua, Inc. (Xi'an, China), which included data pertaining to 22 women and 39 men and assessed 11 normal bone tissues and 50 OS tissues. All tissues were processed as previously described.^21^ Subsequently, tissue sections were collected onto slides, deparaffinized and hydrophilized, and stained with diaminobenzidine for 2 min, followed by counterstaining with hematoxylin (Solarbio, China).^12^ To ensure robustness of results, the sections were then independently examined by two pathologists who were blinded to clinical information. The study has been approved by the research ethics review committee of Jiangxi Provincial People's Hospital, and the approval number is 2021‐032. Due to no provision of clinical samples, our study does not necessitate the completion of an informed consent form.

### 2.4 Cell culture and lentivirus infection

From the Cell Bank of the Chinese Academy of Sciences, four human OS cell types (MG‐63, U‐2OS, HOS, and Saos2) and human osteoblast cells (hFOB1.19) were obtained. All of them were plated on DMEM supplemented with fetal bovine serum, penicillin, and streptomycin, as previously described.^22^

To suppress FAM60A expression in OS cell lines, we used two lentivirus‐mediated si‐FAM60A constructs (si‐FAM60A‐1, CUGACAGUAAACGCUAUGATT; si‐ FAM60A‐2, GCAACCAGAUCAGUAAACUTT), along with a negative control siRNA construct (si‐NC), as previously described.^23^ After transfection of U‐2OS and MG63 cells, objective gene silencing efficiency was also assessed.

### 2.5 RT‐qPCR to assess FAM60A expression in OS

Total RNA was extracted from HOS, U‐2 OS, MG‐63, Saos2, and hFOB1.19 cells, and cDNA synthesis was performed using the following primers: forward, 5′‐CTCCAGTTCTCGATTCACTGAC‐3′ and reverse, 5′‐CGAGTCTCATGCAATCCAAAACA‐3′.^18^ Gene expression was analyzed using the 2^−ΔΔCt^ method with actin as an internal reference.^24^

### 2.6 Western blotting

WB was performed with anti‐FAM60A (ab167180, 1:1000) and actin (1:1000, Abcam, Shanghai, China), as previously described.^25^ Target protein band intensities were quantified with ImageJ, and experiments were independently replicated at least three times.

### 2.7 Cell proliferation and colony formation assays

Cell proliferation rates were analyzed by performing cell counting kit‐8 (CCK‐8) assay. After adding CCK‐8 reagent to each well, U‐2 OS and MG63 cell vitality was examined every 24 h (at 24, 48, 72, and 96 h). Optical density was measured at 450 nm using a microplate reader; a proliferation curve was constructed, and every sample was assayed thrice, as previously reported.^26^ To evaluate colony formation, MG‐63 and U‐2 OS cells were inoculated in a six‐well plate. Subsequently, cultures were fixed and stained, and cells were counted, as previously described.^21^

### 2.8 Cell apoptosis analysis

Cell apoptosis was detected using the Annexin V‐FITC/PI apoptosis detection kit. MG‐63 and U‐2 OS cells were transfected with si‐NC, si‐FAM60A‐1, or si‐FAM60A‐2, and subsequently, they were resuspended and incubated by centrifugation for 30 min. Apoptotic cells were then quantified by a FACScan Flow Cytometer (Becton–Dickinson).^27^

### 2.9 Cell cycle analysis

MG63 and U‐2 OS cells were collected, inoculated in a six‐well plate, and washed with PBS. Subsequently, 0.25% trypsin was added to facilitate cell digestion; when they became round and some were suspended, they were centrifuged at 440 *g* for 5 min. The supernatant thus obtained was discarded, and 500 μL cold ethanol with a volume fraction of 75% was used to fix cells at 4°C. After two PBS washes, fixed cells were resuspended in 500 μL of dyeing working solution (1:9 volume ratio of RNase A/Pi working solution), followed by incubation at room temperature in the dark for 30–60 min. Finally, using flow cytometry and ModFit, cell cycle distribution was evaluated.^28^

### 2.10 Identification of FAM60A co‐expressed genes and upstream transcription factors of FAM60A

FAM60A co‐expressed genes were identified from FAM60A positive relative‐genes (FAM60A‐RGs) and upregulated differentially expressed genes (up‐DEGs) in the included datasets. The correlation between gene expression and FAM60A expression was examined in each included dataset, and genes with Pearson's *r* ≥ 0.3 and *p* < 0.05 in at least six datasets were identified as FAM60A‐RGs. On the other hand, up‐DEGs were identified using the limma package in R.^29^, ^30^ Specifically, for microarray datasets, we chose the limma package; the voom method in the limma package was used to analyze count data from RNA‐seq. Subsequently, DEGs were identified via the limma package, with the criteria being *p* < 0.05 and log_2_ FC ≥1. DEGs appearing in ≥5 datasets were selected as FAM60A‐DEGs. FAM60A co‐expressed genes were defined as those meeting screening requirements of both FAM60A‐RGs and up‐DEGs. The Cistrome Data Browser is a database that can be used to predict transcription factors for target genes based on experimental data from chromatin immunoprecipitation sequencing (ChIP‐Seq).^31^ In this study, we screened and visualized the transcription factors that may regulate FAM60A based on data obtained from only OS tissue or cell lines (MG63, U2OS, 143B, etc.) as the biological sources.

### 2.11 Enrichment analysis

After identifying FAM60A co‐expressed genes, the R package clusterProfiler was used for Kyoto Encyclopedia of Genes and Genomes (KEGG) pathway analysis. STRING (https://string‐db.org/) was used to construct a protein–protein interaction (PPI) network of FAM60A co‐expressed genes. Subsequently, the CytoHubba plugin of Cytoscape 3.8.0 was used to screen hub genes in the PPI network.

### 2.12 Statistical analysis

Between the OS and non‐cancer groups, FAM60A expression was compared using an independent *t*‐test. Cochran's Q test and I^2^ were employed to assess heterogeneity. A random‐effects model was applied if *p* < 0.05 or I^2^ > 50%; in all other scenarios, a fixed‐effects model was applied. Sensitivity analysis was performed to determine the robustness of each analyzed dataset in the meta‐analysis. To determine the presence of publication bias in the included datasets, Begg, Egger, and Deeks' tests were applied. In addition, survival curves were plotted using the Kaplan–Meier method; the log‐rank test was utilized to evaluate survival disparities. *p* < 0.05 indicated statistical significance, unless stated otherwise.

## 3 RESULTS

### 3.1 mRNA expression levels of FAM60A in OS public databases

**FIGURE 1:**
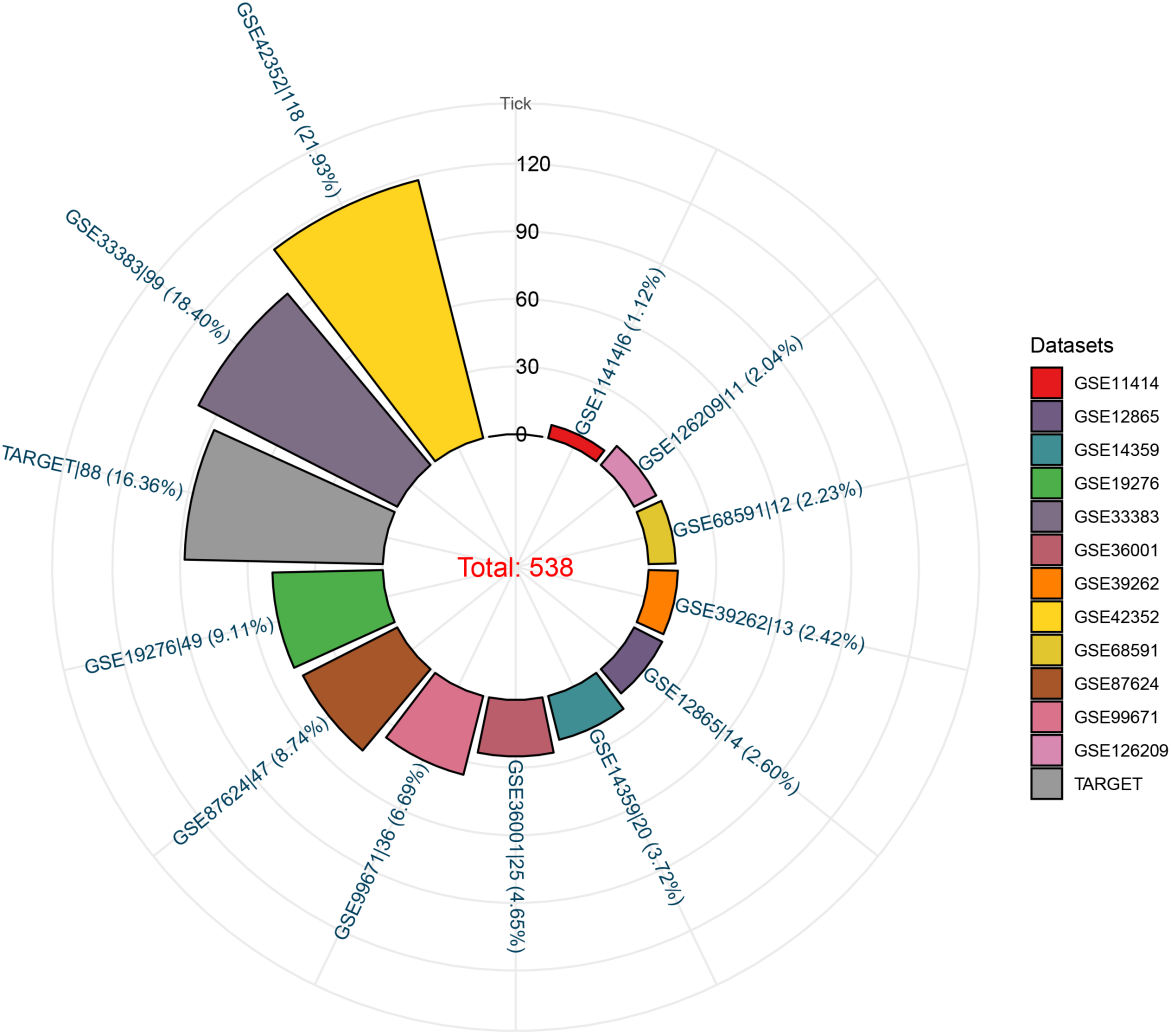
Datasets included in the study and their sample numbers.

**FIGURE 2:**
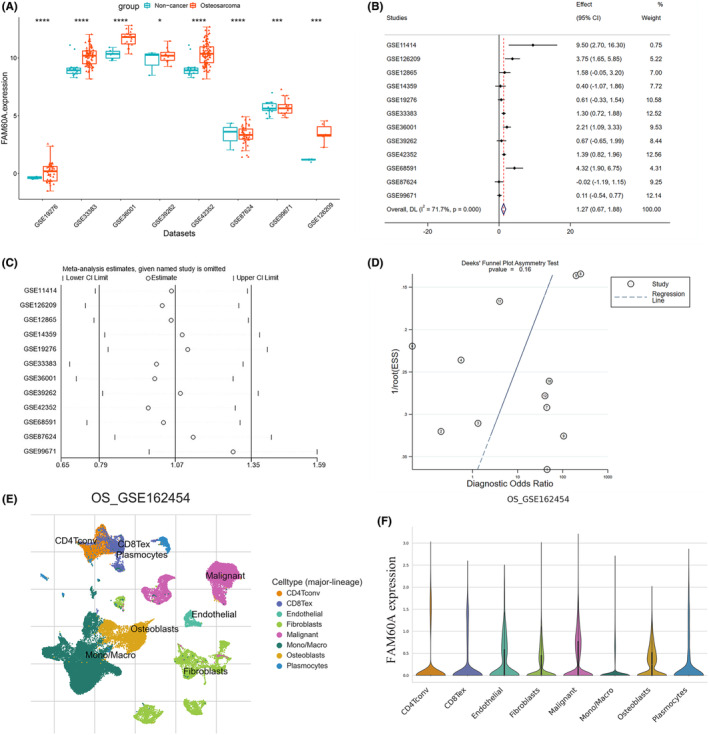
The mRNA expression level and discrimination potential FAM60A in OS. (A) Box plots of FAM60A expression in OS. (B) Forest diagram of FAM60A mRNA expression in OS. (C) Sensitivity analysis of FAM60A expression in OS. (D) Deek's funnel diagram, which suggested no publication bias (*p* > 0.05). (E) Different cell types of OS microenvironment. (F) Expression levels of FAM60A in different types of cells in OS and malignant cells have the highest level of expression among them. FAM60A, Family of homology 60A; OS, osteosarcoma.

On integrating 12 GEO datasets that met our inclusion/exclusion criteria and the TARGET dataset, we obtained 538 samples, including 460 OS and 78 non‐cancer samples. Sample numbers of the included datasets are sequentially listed in Figure 1. Independent *t*‐test results showed that FAM60A was differentially expressed in all included datasets; however, its expression was downregulated in two datasets: GSE87624 and GSE99671 (Figure 2A). Therefore, we integrated FAM60A expression level across all datasets and found that OS samples had considerably higher FAM60A mRNA expression levels than non‐cancer samples (standardized mean difference = 1.27, 95% CI [0.67–1.88], *p* < 0.001); because of the high heterogeneity of final results (I^2^ = 71.7%), a random‐effects model was used for evaluation (Figure 2B). According to the subgroup analysis based on dataset type, extremely high heterogeneity was present in both the RNA‐seq data and microarray data (I^2^ = 81.7% and 57.3%, respectively). Thus, the observed heterogeneity was derived from the dataset itself rather than the data type. Subsequent sensitivity analyses indicated that individual datasets were not the source of heterogeneity (Figure 2C and Figure S1). Further, Begg, Egger, and Deeks' tests did not reveal any substantial publication bias (Figure 2D, Figure S2 and S3). Based on data deposited in the TISCH2 database, we investigated the degree of FAM60A expression in different cell types according to the OS microenvironment (Figure 1E). The results showed that FAM60A had the highest expression level in OS malignant cells, as shown in Figure 1F.

**FIGURE 3:**
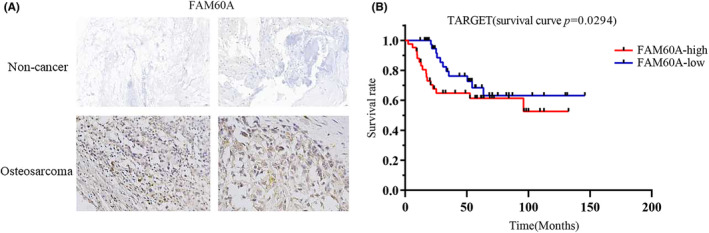
The protein levels and prognostic significance of FAM60A in OS patients. (A) FAM60A expression was intense in OS tissues compared to non‐cancer tissues. (B) FAM60A high expression was related to shorter survival time of OS patients based on TARGET dataset (*p* = 0.0294). FAM60A, family of homology 60A; OS, osteosarcoma; TARGET, Therapeutically Applicable Research To Generate Effective Treatments.

### 3.2 Clinical significance of FAM60A in patients with OS

As described above, our analyses revealed high mRNA expression levels of FAM60A in OS samples. IHC indicated overall light blue staining for FAM60A in normal tissues (upper panel, Figure 3A). These staining results indicate that its expression level in non‐cancer tissues was downregulated. On the contrary, obvious overall brown staining for FAM60A was observed in OS tissue (lower panel, Figure 3A). This indicates that its expression level was upregulated in OS tissues. The above results demonstrate that OS samples showed significantly higher protein expression levels of FAM60A than normal tissue samples.

Further, we integrated the expression and prognostic data for FAM60A in TARGET, and used the median expression of FAM60A (2.466) in OS patients as the criterion for dividing the patients into a high expression group (*n* = 42) and low expression group (*n* = 43) for survival analysis. The results of Kaplan–Meier survival analysis indicated that higher FAM60A expression often predicted worse prognosis in patients with OS (Figure 3B). These results were confirmed in the R2 database (https://hgserver1.amc.nl/cgi‐bin/r2/main.cgi), and the outcomes, likewise, demonstrated that high FAM60A expression was a poor prognostic factor for OS (Figure S4).

### 3.3 Expression of FAM60A in OS cell lines

**FIGURE 4:**
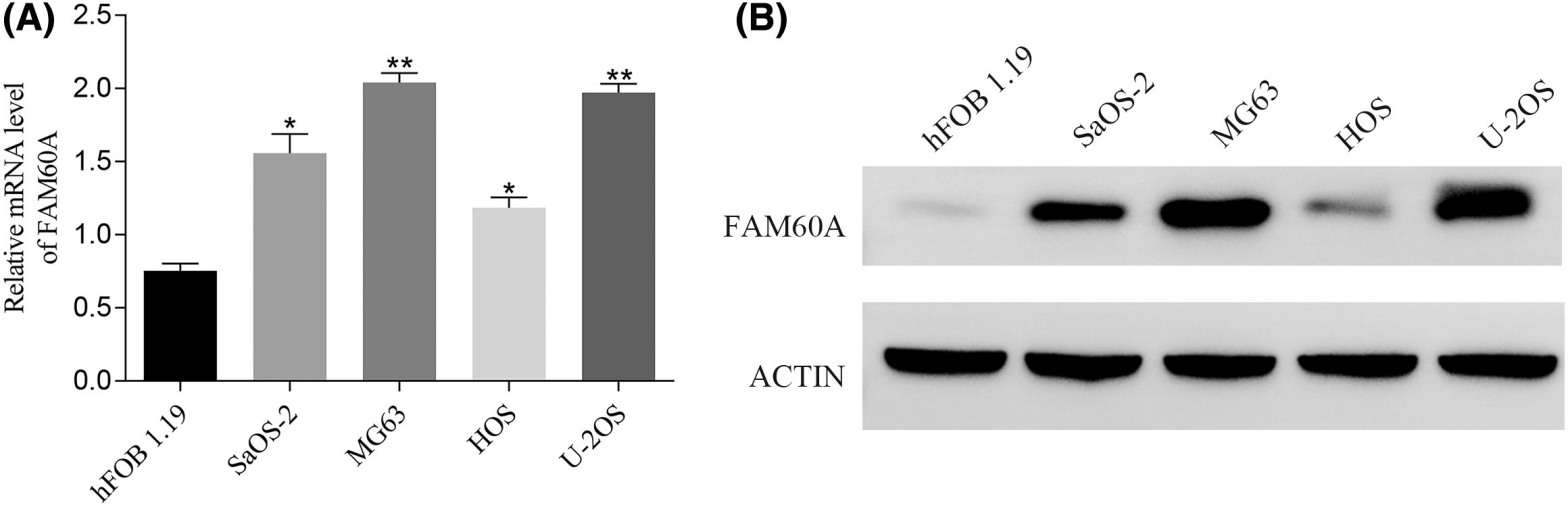
The mRNA and protein expression level of FAM60A in OS cell lines. (A) RT‐qPCR determined FAM60A mRNA expression in human OS cell lines (Saos‐2, MG63, HOS, and U‐2OS) was higher than that in human osteoblast cells (hFOB1.19). (B) WB assay determined FAM60A protein expression in human OS cell lines (Saos‐2, MG63, HOS and U‐2OS) was higher than that in human osteoblast cells (hFOB1.19), the raw data with detail description were shown in additional Figure S5. FAM60A, family of homology 60A; OS, osteosarcoma; RT‐qPCR, real‐time quantitative PCR; WB, western blot; *p < 0.05, **p < 0.01, ***p < 0.001 versus hFOB1.19.

FAM60A expression levels in OS cell lines were determined by performing RT‐qPCR and WB. Relative to hFOB1.19 cells, SaOS‐2, MG63, HOS, and U‐2 OS cells showed remarkably higher mRNA and protein expression levels of FAM60A (Figure 4A,B). This indicated that FAM60A expression was stably elevated in patients with OS and in OS cell lines, suggesting the involvement of FAM60A in OS progression.

### 3.4 Effects of downregulation of FAM60A expression on OS cell proliferation

**FIGURE 5:**
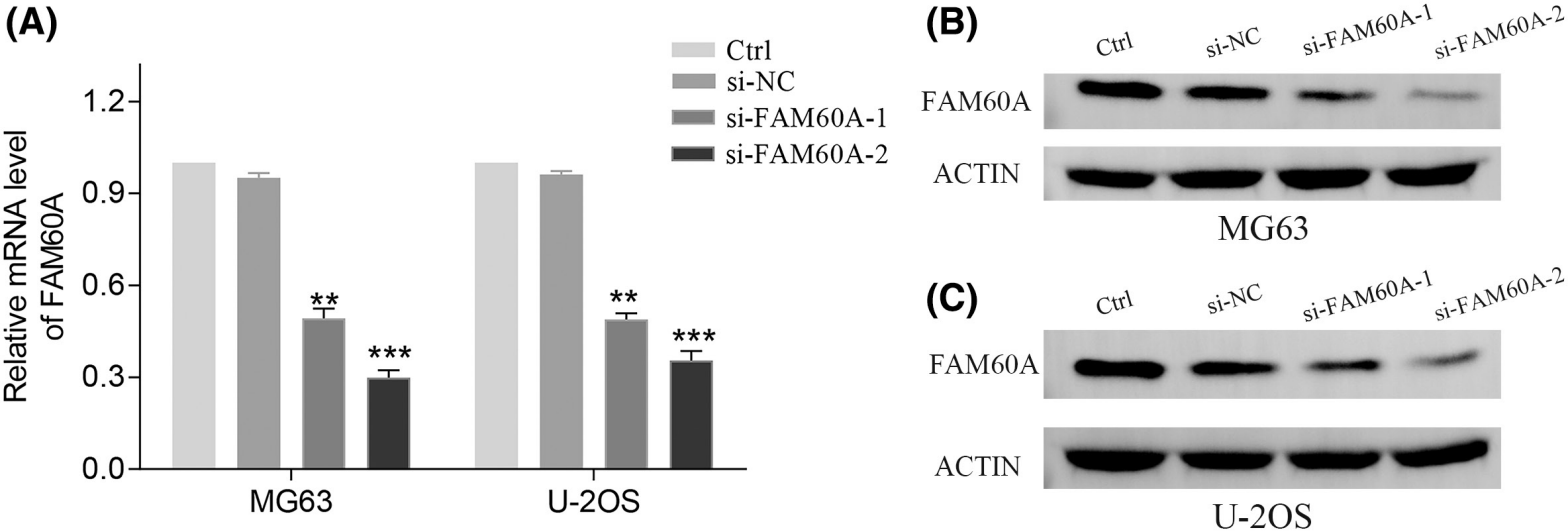
The determination of FAM60A silencing efficiency in OS cell lines. (A) The mRNA expression level of FAM60A in MG63 and U‐2OS cell lines after targeted knockdown determined by RT‐qPCR. (B) The protein expression level of FAM60A in MG63 cell lines after targeted knocking down determined by WB assay, si‐FAM60A‐2 sites showed the most prominent knockdown effect, the raw data with detail description were shown in additional Figure S6. (C) The protein expression level of FAM60A in U‐2OS cell lines after targeted knockdown determined by WB assay, si‐FAM60A‐2 sites showed the most prominent knockdown effect, the raw data with detail description was shown in additional Figure S7. FAM60A, family of homology 60A; OS, osteosarcoma; RT‐qPCR, real‐time quantitative PCR; WB, western blot; *p< 0.05, **p < 0.01, ***p < 0.001 versus hFOB1.19 or si‐NC.

**FIGURE 6:**
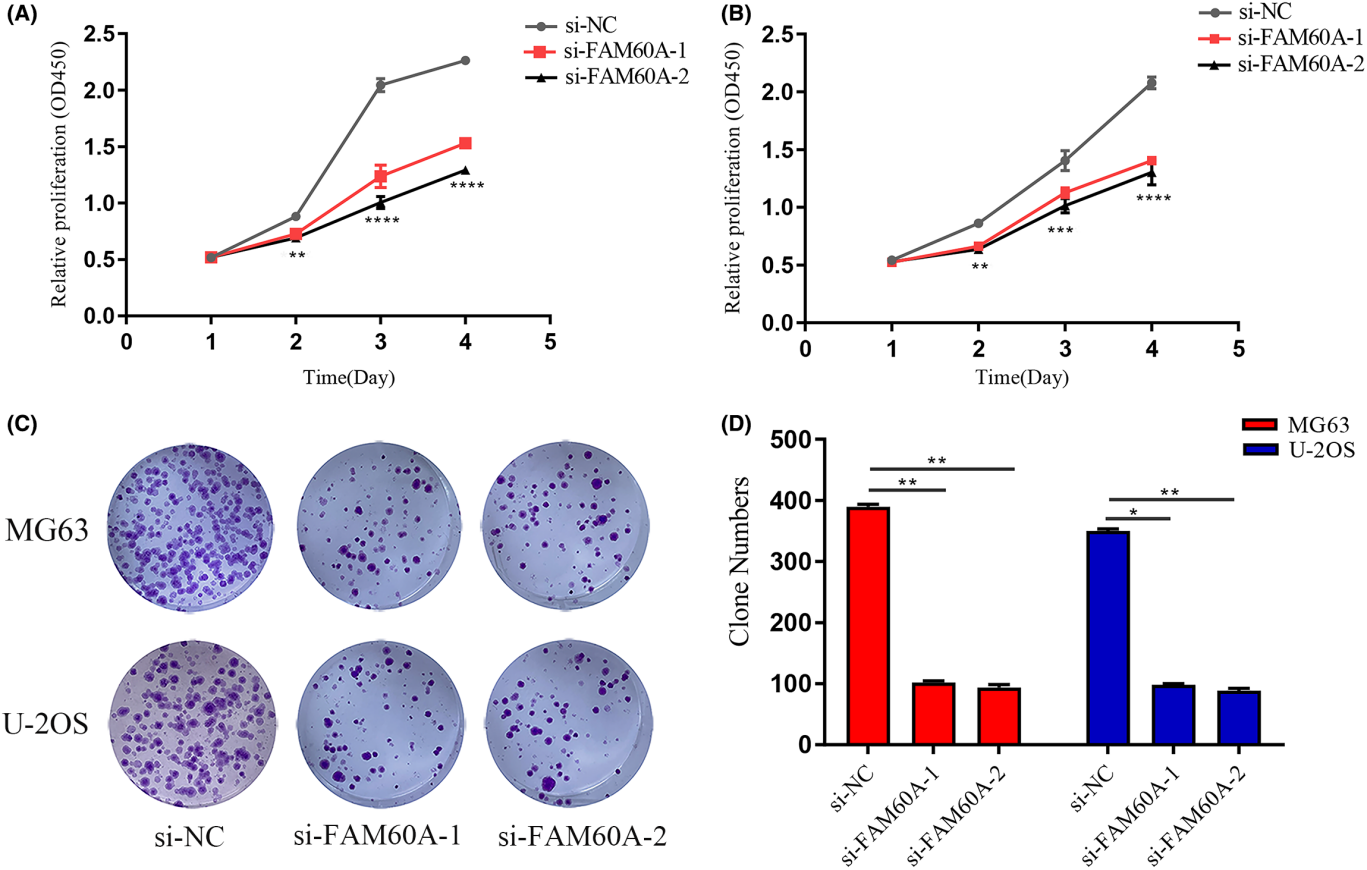
The effects of downregulation of FAM60A in proliferation and colony formation of OS cells. (A) CCK8 assay analysis showed that silencing FAM60A inhibited the proliferation of MG63 cell lines. (B) CCK8 assay analysis showed that silencing FAM60A inhibited the proliferation of U‐2OS cell lines. (C‐D) Silencing FAM60A impaired colony forming ability in MG63 and U‐2OS cell lines. FAM60A, family of homology 60A; OS, osteosarcoma; CCK8, Cell Counting Kit‐8; *p < 0.05, **p < 0.01, *** *p* < 0.001 versus si‐NC.

In comparison to SaOS‐2 and HOS cells, U‐2 OS and MG63 cells showed higher mRNA and protein expression levels of FAM60A. To investigate the role of FAM60A in OS cells, we knocked down the expression of FAM60A in U‐2 OS and MG63 cells. The difference in the expression levels of FAM60A in U‐2 OS and MG63 cells versus those in the blank and negative control (si‐NC) groups was compared by RT‐qPCR and WB; transfection efficiency was also verified. We found that si‐FAM60A transfection significantly reduced mRNA expression levels of FAM60A in U‐2 OS and MG63 cells (Figure 5A). Moreover, in MG63 and U‐2OS cells, both siRNA‐2 sites showed the most prominent knockdown effect (Figure 5B,C). Furthermore, we evaluated the effect of silencing FAM60A on the proliferative capacity of OS cells by performing CCK‐8 and colony formation assays. In comparison with the si‐NC group, silencing FAM60A was found to efficiently suppress cell proliferation and colony formation ability of MG63 and U‐2 OS cells, suggesting that FAM60A promotes the proliferation of OS cells (Figure 6A–D).

### 3.5 Effects of downregulation of FAM60A expression on OS cell apoptosis and cell cycle

**FIGURE 7:**
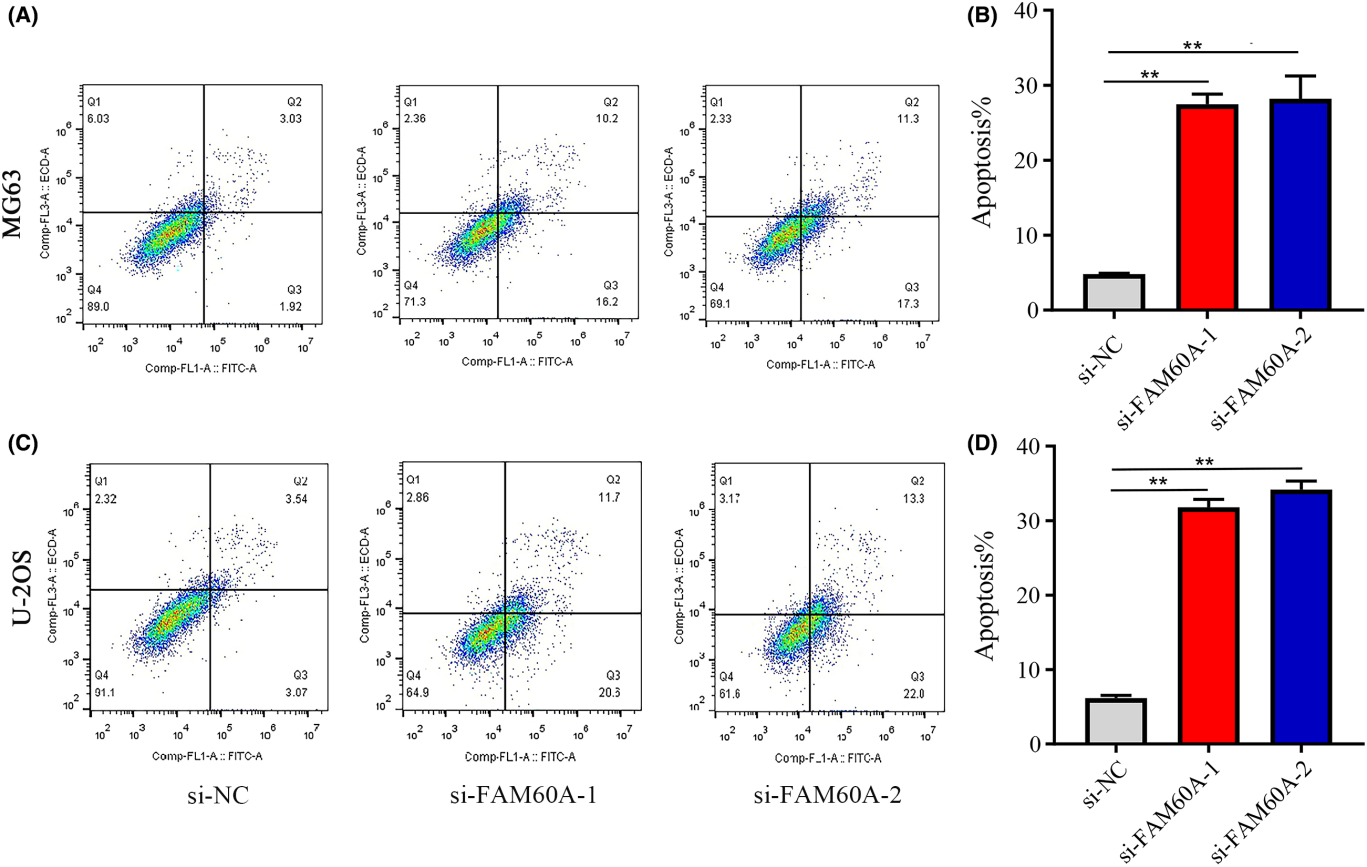
The effects of downregulation of FAM60A in cell apoptosis of OS cells. (A, B) Apoptosis tests showed that silencing FAM60A promoted the apoptosis of the MG63 cell line, si‐FAM60A‐2 sites showed the most prominent apoptosis effect. (C, D) Apoptosis tests showed that silencing FAM60A promoted the apoptosis of the U‐2OS cell line, si‐FAM60A‐2 sites showed the most prominent apoptosis effect. FAM60A, family of homology 60A; OS, osteosarcoma; *p < 0.05, **p < 0.01, *** *p* < 0.001 versus si‐NC.

Flow cytometry was used to analyze the effects of knocking down FAM60A expression levels on OS cell apoptosis. We found that relative to the si‐NC group, MG63 and U‐2 OS cells transfected with si‐RNA showed higher cell apoptosis rates; si‐FAM60A‐2‐transfected cells showed the highest cell apoptosis rates (Figure 7A–D).

**FIGURE 8:**
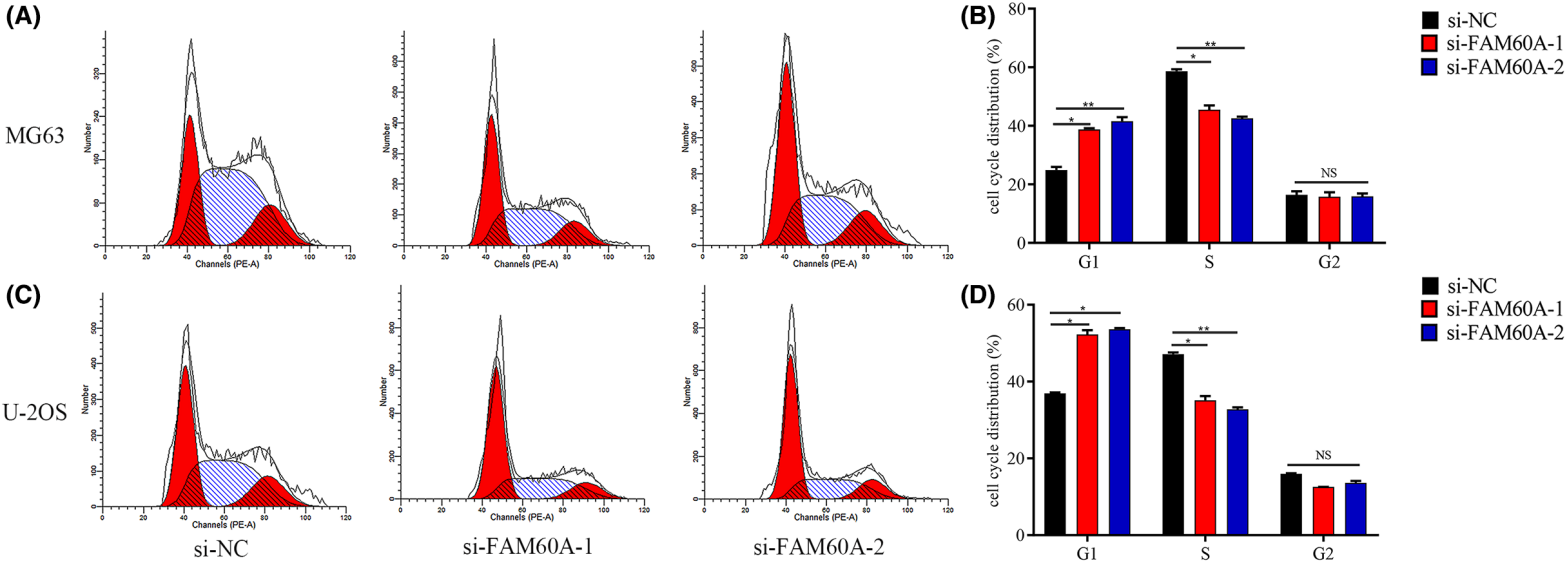
The effects of downregulation of FAM60A in cell cycle of OS cells. (A, B) Percentage of each phase of the cell cycle were determined by using flow cytometry in MG63 cell line after targeted knocking down FAM60A. (C, D) Percentage of each phase of the cell cycle were determined by using flow cytometry in U‐2OS cell line after targeted knocking down FAM60A. FAM60A, family of homology 60A; OS, osteosarcoma; *p < 0.05, **p < 0.01, *** *p* < 0.001 versus si‐NC.

Flow cytometry was used to assess cell cycle distribution so as to determine whether FAM60A participates in cell cycle progression. The ratio of G1 phase cells showed an increase within siRNA1‐FAM60A and siRNA2‐FAM60A groups of MG63 or U‐2 OS cells, while that of S phase cells showed a significant decrease; however, neither MG63 nor U‐2 OS cells showed considerable differences in the fraction of G2 phase cells (Figure 8A–D).

### 3.6 Functional enrichment analysis

**FIGURE 9:**
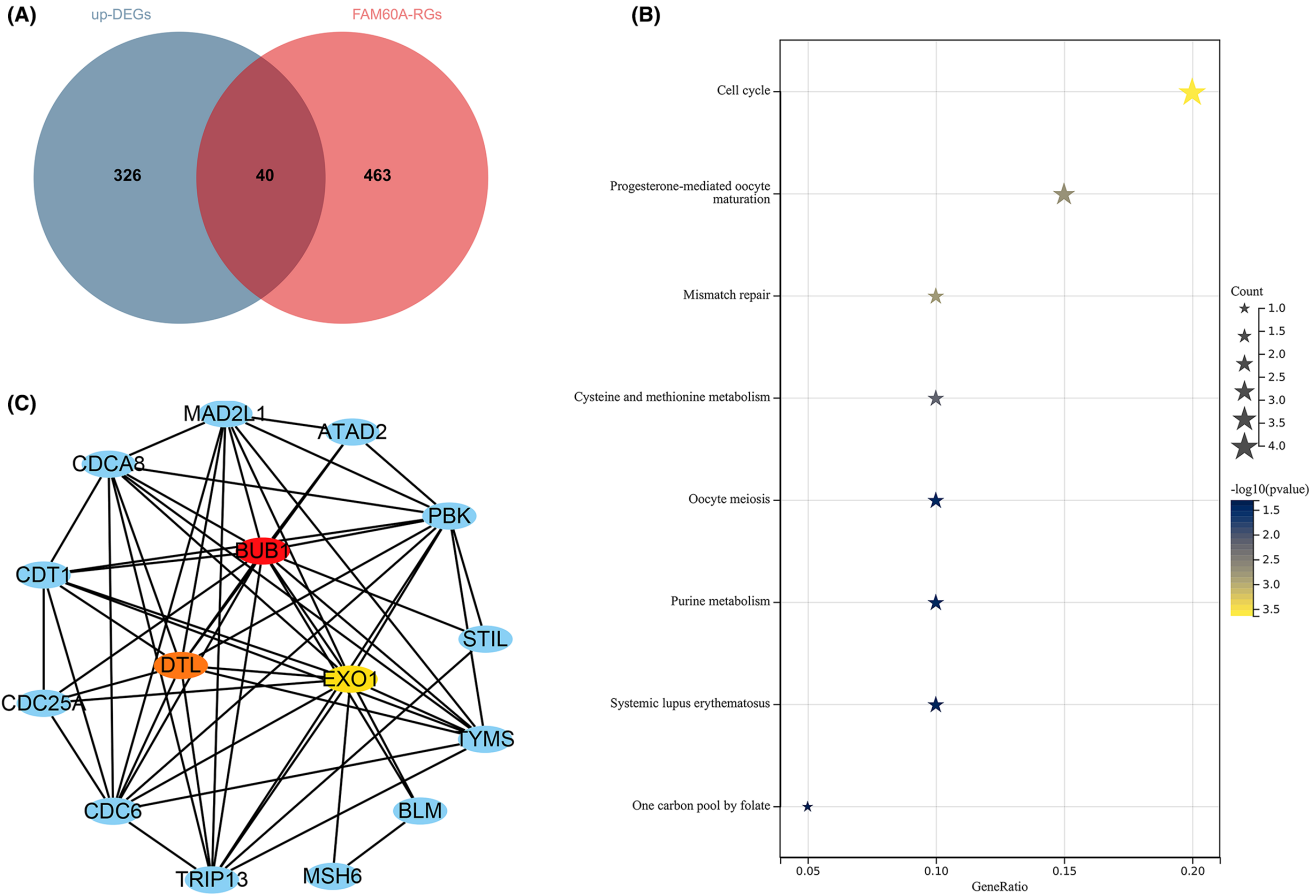
Functional enrichment and PPI analysis. (A) Venn diagram showing the FAM60A co‐expressed genes in OS based on up‐DEGs and FAM60A‐RGs. (B) Enrichment terms of FAM60A co‐expressed genes in KEGG pathway. (C) PPI network analysis showed that BUB1, DTL, and EXO1 had the top three degrees of connection of the FAM60A co‐expressed genes. DEGs, differentially expressed genes; FAM60A, Family of homology 60A; OS, osteosarcoma; PPI, protein–protein interaction; RGs, positive relative‐genes.

Overall, 366 up‐DEGs and 503 FAM60A‐RGs were selected from the included datasets, and 40 FAM60A co‐expressed genes were identified from the intersection (Figure 9A). The enrichment analysis of these co‐expressed genes highlighted the potential molecular mechanism of FAM60A in OS. KEGG pathway analyses suggested that the cell cycle pathway was the most prominent, corresponding to our results pertaining to OS cell cycle analysis. Collectively, these findings indicated that FAM60A seems to play a key role in OS cell cycle progression (Figure 9B, Table 1). In addition, we noticed significant ChIP‐seq peaks near the FAM60A transcription start site; this indicates that the transcription factor MYC may act as an upstream regulator of FAM60A in OS (Figure S8A). We also investigated the interaction between MYC and FAM60A in the prognosis of OS patients. Based on TARGET dataset, the overexpression of MYC is closely related to the poor prognosis of patients with OS (HR = 2.40, 95% CI [1.08–5.35], *p* = 0.03) (Figure S8B), furthermore, we identified patients with upregulation of FAM60A expression (as mentioned earlier) to conducted survival analysis, the result indicated that MYC is also a poor prognostic factor and has a higher risk ratio compared to patients with low expression of FAM60A (HR = 3.04, 95% CI [1.07–8.66], *p* = 0.03) (Figure S8C), which may indicate that FAM60A is regulated by the transcription factor MYC and jointly participates in the malignant progression of OS, and the higher the expression level of both, the worse the clinical outcome of OS patients.

**TABLE 1:** KEGG enrichment analysis of FAM60A co‐expressed genes.

Term	Description	*p*‐value	Count
hsa04110	Cell cycle	2.29E‐04	4
hsa03430	Mismatch repair	1.49E‐03	2
hsa04914	Progesterone‐mediated oocyte maturation	1.80E‐03	3
hsa00270	Cysteine and methionine metabolism	6.65E‐03	2
hsa04114	Oocyte meiosis	4.02E‐02	2
hsa00230	Purine metabolism	4.19E‐02	2
hsa05322	Systemic lupus erythematosus	4.37E‐02	2
hsa00670	One carbon pool by folate	4.94E‐02	1

Abbreviations: FAM60A, family of homology 60A; KEGG, Kyoto Encyclopedia of Genes and Genomes.

### 3.7 PPI network analysis

To identify key genes that closely interact with FAM60A, we used STRING to build a PPI network; the default parameter was interaction score of 0.4. The output was then imported into Cytoscape, and key genes in the PPI network were filtered by the MCC method utilizing the CytoHubba plugin. Ultimately, the three highest‐scoring genes, namely BUB1, DTL, and EXO1, were identified as key genes, and were believed to closely interact with FAM60A (Figure 9C). Furthermore, we verified the expression levels of these three genes and found that they were highly expressed in OS samples (Figure S9A–C).

## 4 DISCUSSION

OS is associated with a high risk of metastasis and recurrence, chemotherapy resistance, and poor prognosis; thus, elucidating the underlying pathogenesis and identifying new biological markers for early diagnosis and targeted therapy are pivotal.^32^ FAM60A, a subunit of the SIN3A/HDAC complex, is a cell cycle regulatory protein with a key role in the division of malignant tumors.^9^ The downregulation of its expression in esophageal cancer cells has been found to reduce the number of G1 phase cells, prevent the entry of cells into the G2/M phase, inhibit cell proliferation, promote apoptosis, and inhibit metastasis and invasion in vitro.^12^, ^33^, ^34^ Moreover, *H. pylori* infection was reported to increase the ratio of gastric cancer cells in the S phase and reduce the proportion of those in the G1/G0 phase; the knockdown of FAM60A expression rescued this phenotype and ultimately caused G1/G0 phase arrest.^17^, ^25^ Some scholars have pointed out that FAM60A can act as a tumor promoter in pancreatic cancer and mediate malignant behaviors such as proliferation and invasion. Depletion of FAM60A impairs the activities of Akt and β‐catenin, weakens the development of pancreatic cancer cells after xenotransplantation, and increases the sensitivity of pancreatic cancer cells to gemcitabine; thus, it may be a new therapeutic target for pancreatic cancer.^35^ Collectively, according to previous studies, FAM60A plays different roles in different cancers. However, to date, no study has reported the presence of a relationship between FAM60A and OS; consequently, the significance of FAM60A in OS remains uncertain.

Herein on analyzing 538 OS samples from public databases, we found FAM60A expression to be significantly elevated in OS, and the highest expression level in malignant tumor cells of OS. Besides, FAM60A overexpression was associated with poor prognosis in patients with OS. By collecting tissue microarrays, the protein expression level of FAM60A was found to be upregulated in patients with OS. Further, on knocking down FAM60A expression in MG63 and U‐2OS cells, we realized that the downregulation of FAM60A expression inhibited the proliferation of OS cells, induced cell apoptosis, increased the ratio of G1 phase cells, and blocked cell entry into the S phase. We also performed functional enrichment analyses of FAM60A co‐expressed genes to elucidate the molecular mechanism underlying the action of FAM60A in OS, and found that “cell cycle” was the most prominently enriched pathway, consistent with the results of our cell cycle analysis, suggesting that FAM60A affects OS development by participating in OS cell cycle.

PPI network analyses yielded three hub genes, namely BUB1, DTL, and EXO1, showing the closest association with FAM60A. In eukaryotes, BUB1, a protein kinase from the SAC family, monitors chromosomal segregation during mitosis, which indicates that BUB1 dysregulation may lead to chromosomal instability and eventually cancer development.^36^ Peng et al. applied computational biology to report that BUB1 expression is upregulated in OS; besides, wet experiments verified that downregulating BUB1 expression inhibited OS cell proliferation, migration, and invasion and stimulated apoptosis.^37^ In vitro investigations into adenocarcinoma of the lung and breast cancer support the notion that BAY 1816032, which was previously thought to be a newly discovered inhibitor of BUB1 catalytic activity, may have therapeutic potential for the treatment of OS. Given that chemotherapy resistance in OS is one of the leading causes of death, BAY 1816032 has a lot of promise as a novel candidate therapeutic agent.^38^ As a retinoid acid‐regulated nuclear matrix‐associated protein, DTL has been shown to be widely involved in cell proliferation, cell cycle, and cell invasion in breast and gastric cancers.^39^, ^40^ An earlier study revealed that miR‐215 caused G2 phase arrest and reduced the proliferation of OS cells by inhibiting DTL expression; in addition, resistance of OS cells to methotrexate was reversed, which suggests that DTL can serve as a potential therapeutic target for OS.^41^, ^42^ On the other hand, Tang et al. examined the association of DTL with numerous immune cells and conducted a pan‐cancer analysis of its expression. The findings demonstrated a positive correlation between the expression of DTL and the infiltration of CD3+ T cells in liver cancer, bladder urothelial carcinoma, and stomach adenocarcinoma. Additionally, on the basis of correlation analysis of the genes included in the follow‐up immunological examination, effective immunotherapy outcomes were anticipated.^43^ Thus, given the significance of immunotherapy in the management of malignant tumors, targeting DTL might provide us with fresh perspectives on OS therapy. EXO1 plays a key role in protecting genome stability during DNA replication and post‐replication processes; further, it participates in and plays important roles in various DNA repair processes.^44^ Investigations using CRISPR technology to create drug‐resistant ovarian cancer cell lines have demonstrated that the absence of EXO1 can reverse drug resistance and improve sensitivity to cisplatin and doxorubicin, and it was therefore identified as a potential therapeutic target.^45^ However, the association between EXO1 and OS remains to be explored. We believe that EXO1 plays a vital role in OS development. The transcription factor MYC is considered to be an upstream regulatory factor of FAM60A, which has been considered to play an important role in regulating the progress and metastasis of OS in recent years, further, the amplification of MYC is closely related to the occurrence and development of OS, especially in children.^46^, ^47^ Based on the results of the ChIP‐Seq experiment in this study, we propose the hypothesis that MYC regulates the expression of FAM60A and enhances the progression of OS.

This study has some limitations: (1) our results showed high heterogeneity (I^2^ = 71.7%), which may weaken the credibility of our conclusions even though the sensitivity analyses did not reveal the source of heterogeneity; in the future, we aim to further explore the expression level of FAM60A in OS through multicenter clinical studies. (2) We found that FAM60A contributes to the development of OS in an oncogenic manner, but animal studies need to be conducted to verify our data. (3) We speculate that FAM60A promotes OS progression via its interaction with BUB1, DTL, and EXO1, and its regulation by MYC. However, the validation of hub genes and upstream transcription factors requires co‐immunoprecipitation experiments and rescue experiments on in vitro cell lines.

## 5 CONCLUSION

We found FAM60A expression to be significantly upregulated in OS and closely associated with worse prognosis. In addition, FAM60A was found to promote OS cell proliferation, inhibit apoptosis, and participate in OS cell cycle regulation. We hypothesize that FAM60A interacts with BUB1, DTL, and EXO1 to promote OS development and progression. Collectively, our results suggest that FAM60A can serve as a new biomarker and therapeutic target for OS.

## AUTHOR CONTRIBUTIONS

**Yu Sun:** Methodology (lead); project administration (equal); resources (equal). **Yu‐Nan Man:** Data curation (equal); formal analysis (lead); visualization (equal). **Jin‐hui Cheng:** Software (lead); supervision (equal). **Jing‐tang Li:** Investigation (equal); resources (equal); software (equal). **Ya‐yun Liu:** Conceptualization (lead); funding acquisition (lead); writing – original draft (equal); writing – review and editing (equal).

## FUNDING INFORMATION

This work was supported by the National Natural Science Foundation of China under Grant [grant number 82160528] and by the General topics of Jiangxi Provincial Science and Technology Department [grant number 20212BAB206059].

## CONFLICT OF INTEREST STATEMENT

The authors declare no conflict of interest.

## ETHIC APPROVAL STATEMENT

Ethical approval is not applicable to this study and the raw data can be obtained from the corresponding author or download from the public database.

## Supporting information


Figure S1.

Figure S2.

Figure S3.

Figure S4.

Figure S5.

Figure S6.

Figure S7.

Figure S8.

Figure S9.

## Data Availability

The datasets generated and analysed during the current study are available in the Therapeutically Applicable Research to Generate Effective Treatments (TARGET) database (www.ocg.cancer.gov/programs/target). The Gene Expression Omnibus (GEO, www.ncbi.nlm.nih.gov/geo/, GSE11414, GSE126209, GSE12865, GSE14359, GSE19276, GSE39262, GSE68591, GSE87624, GSE99671, GSE33383, GSE36001, GSE42352) database.
