# Supplementary figures and images for: FAM60A promotes osteosarcoma development and progression
Source: Cancer Med. 2023 Jul 12;12(16):17491–503. doi: 10.1002/cam4.6343 (PMC10501228; doi:10.1002/cam4.6343)

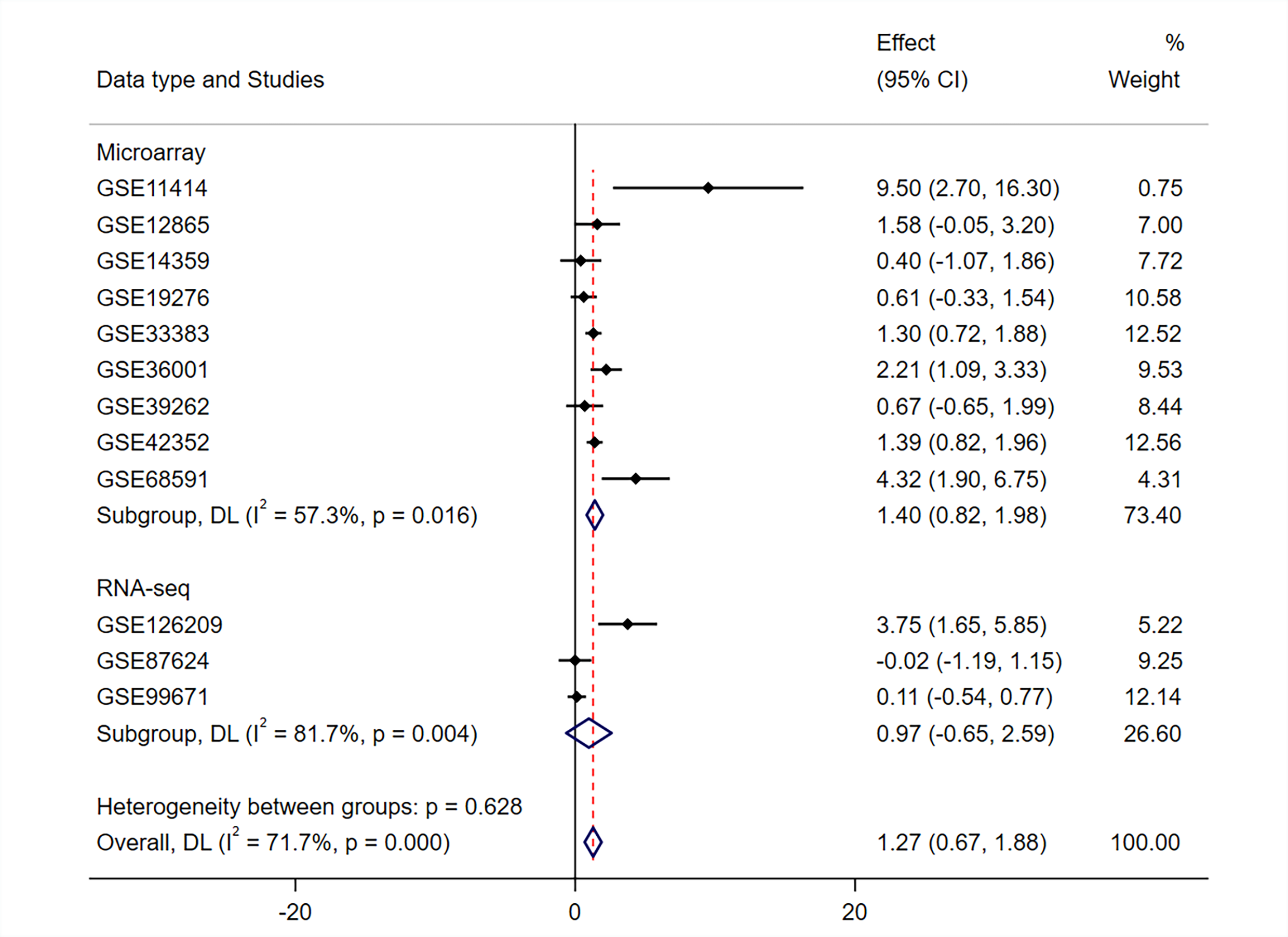

Supplement: Supplementary file 1 — Figure S1. [file CAM4-12-17491-s006.tif]

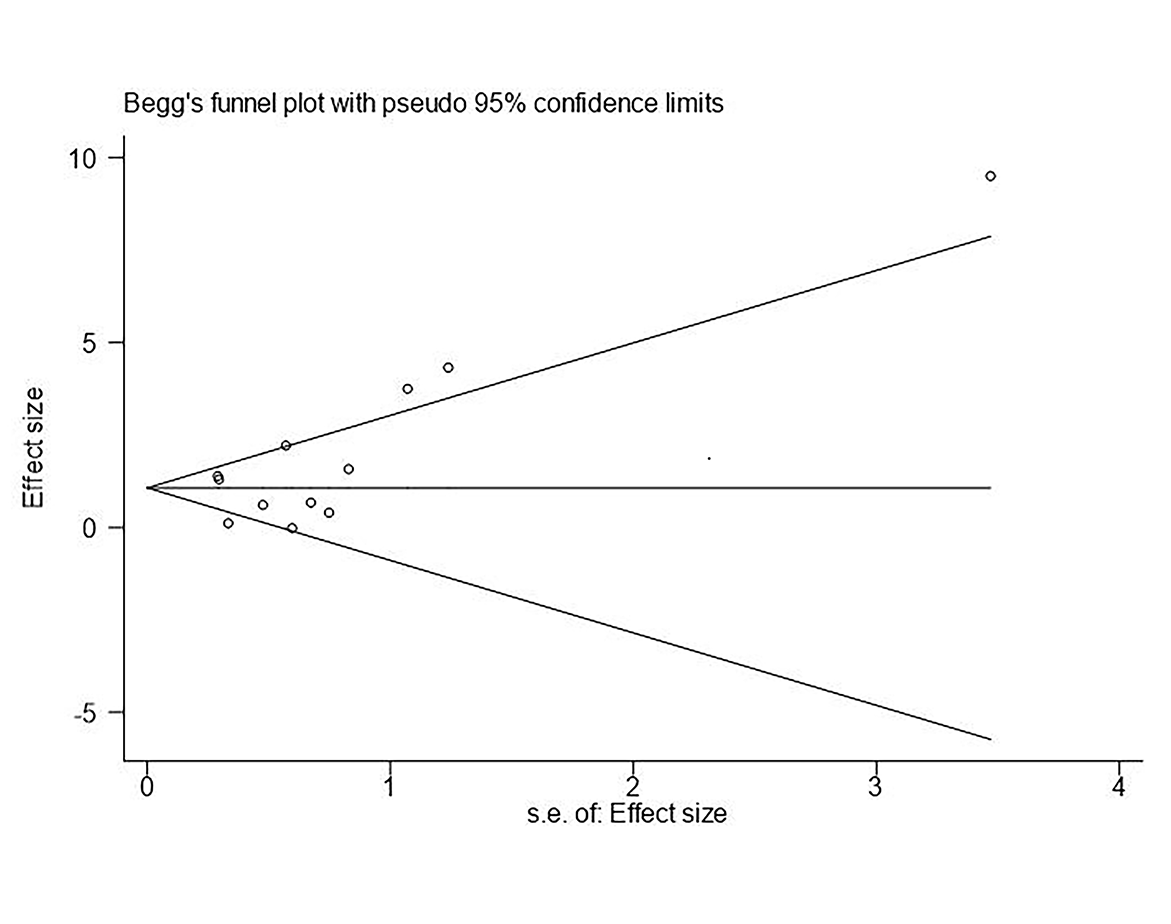

Supplement: Supplementary file 2 — Figure S2. [file CAM4-12-17491-s007.tif]

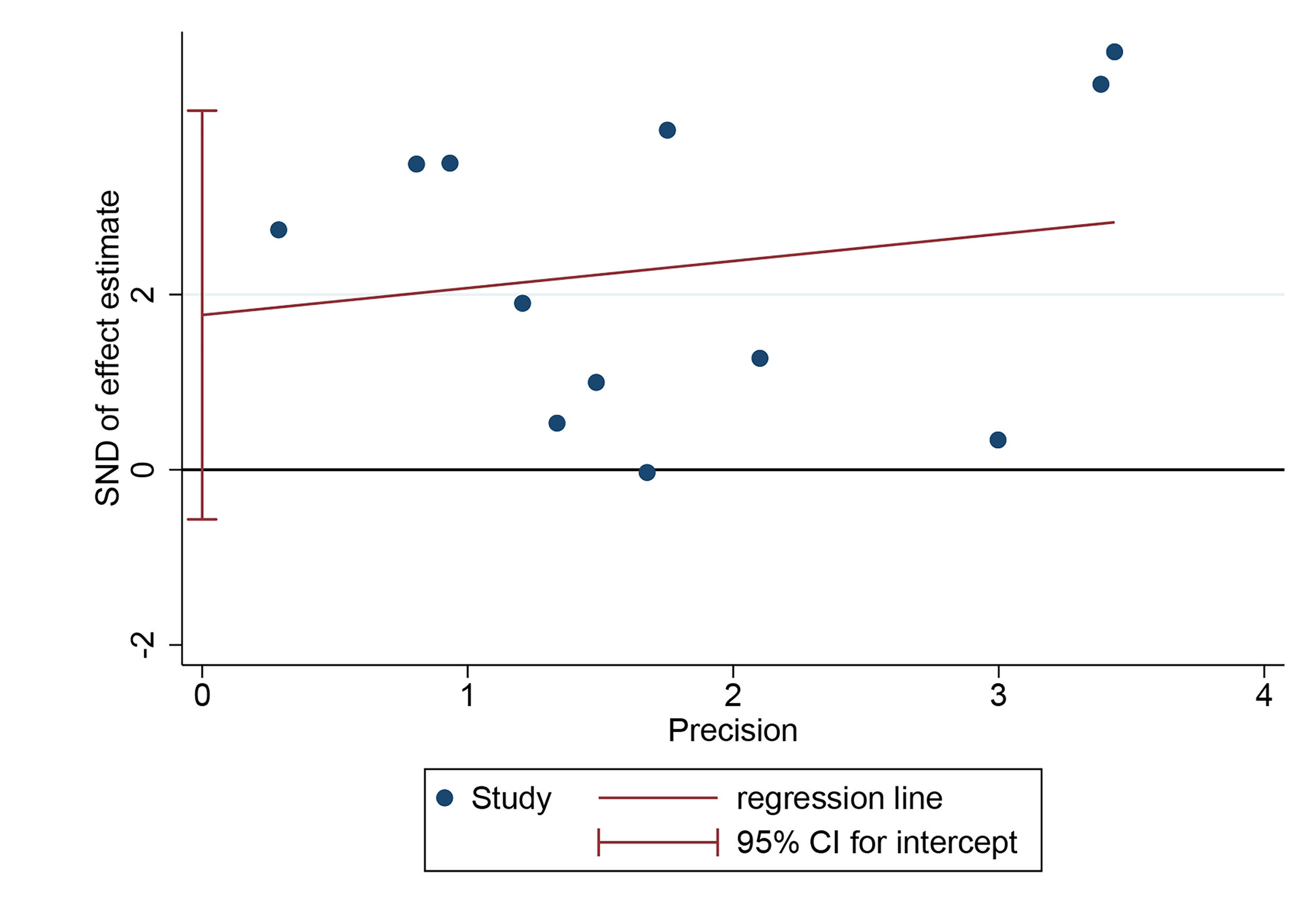

Supplement: Supplementary file 3 — Figure S3. [file CAM4-12-17491-s001.tif]

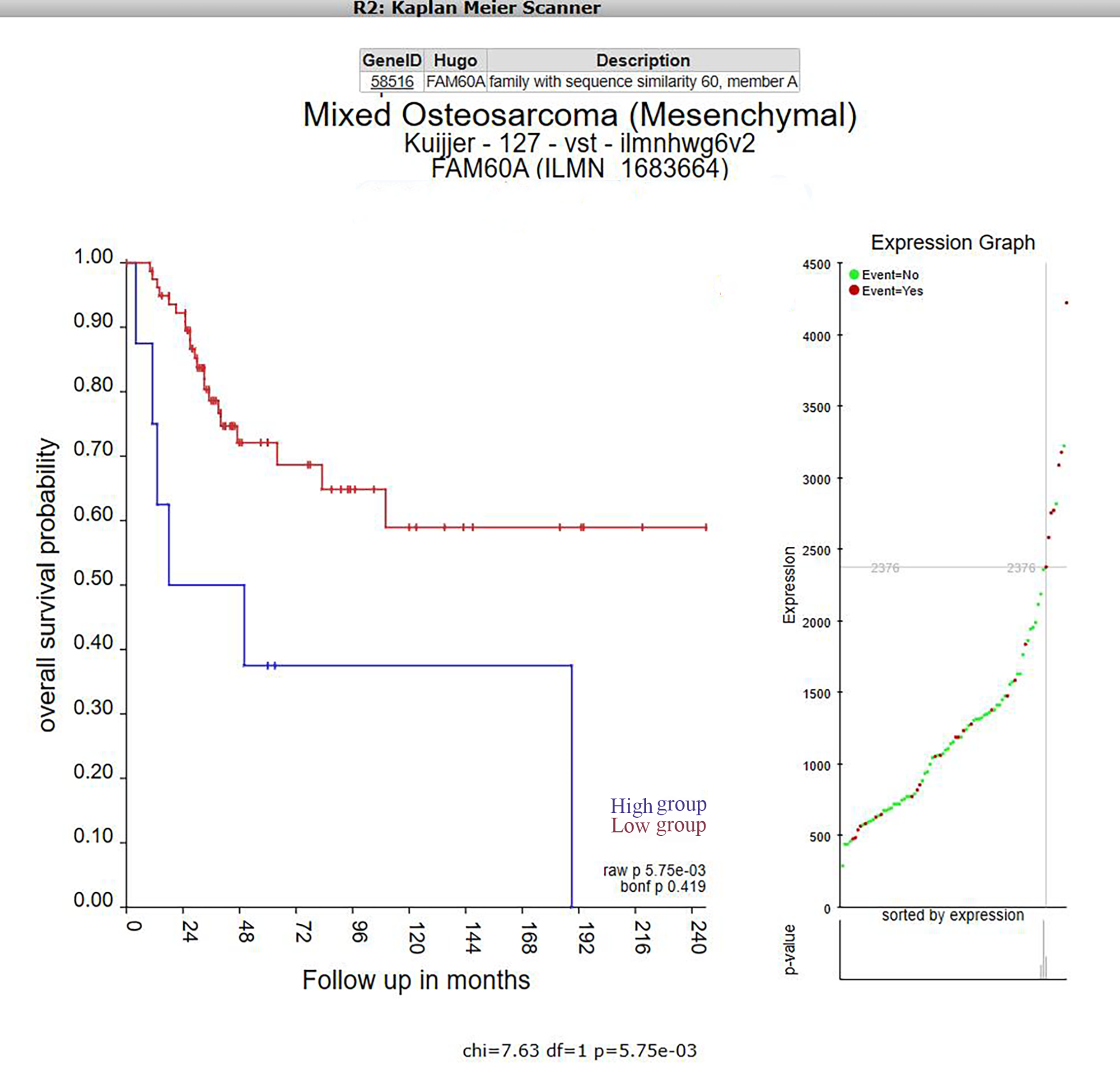

Supplement: Supplementary file 4 — Figure S4. [file CAM4-12-17491-s008.tif]

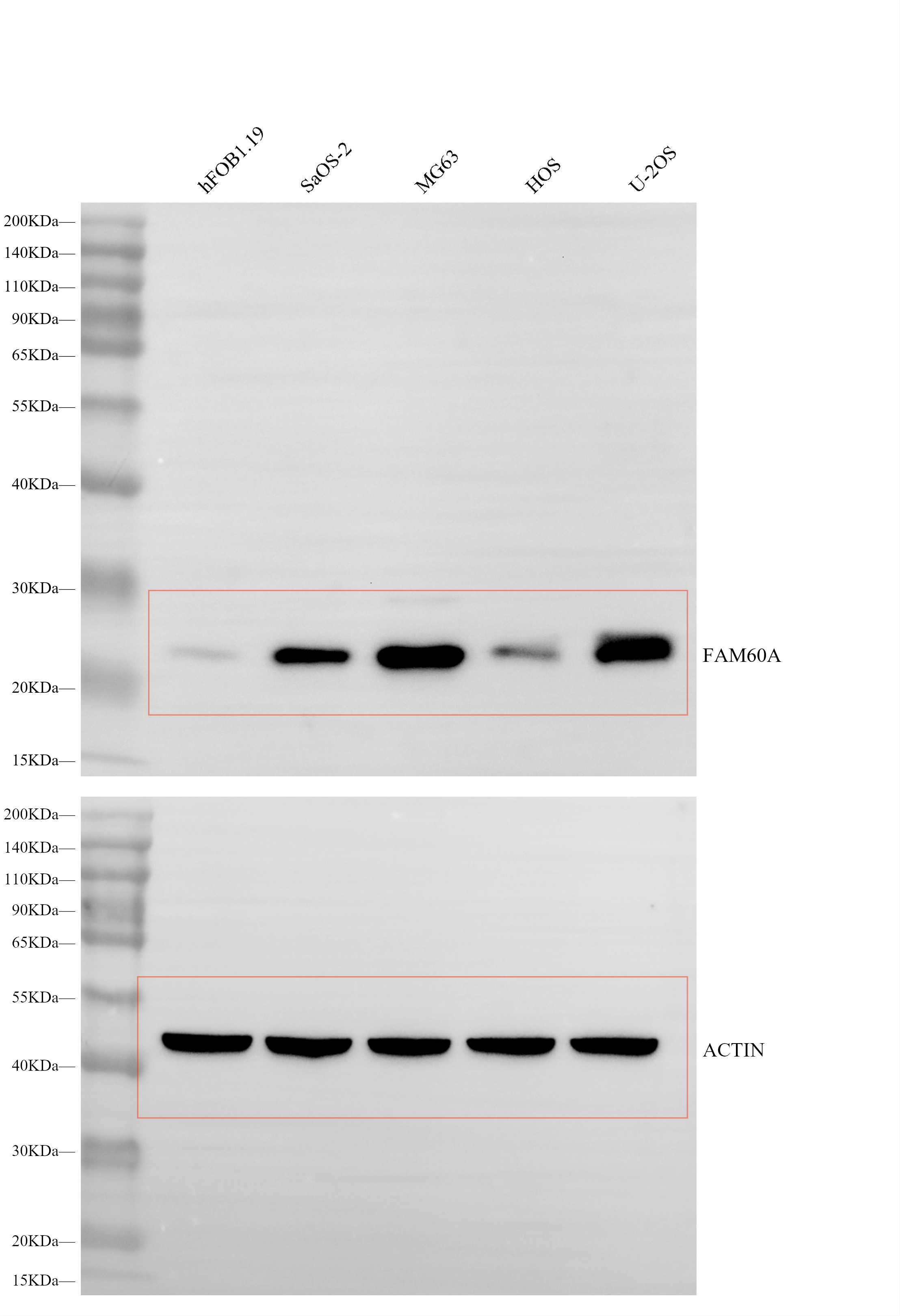

Supplement: Supplementary file 5 — Figure S5. [file CAM4-12-17491-s004.tif]

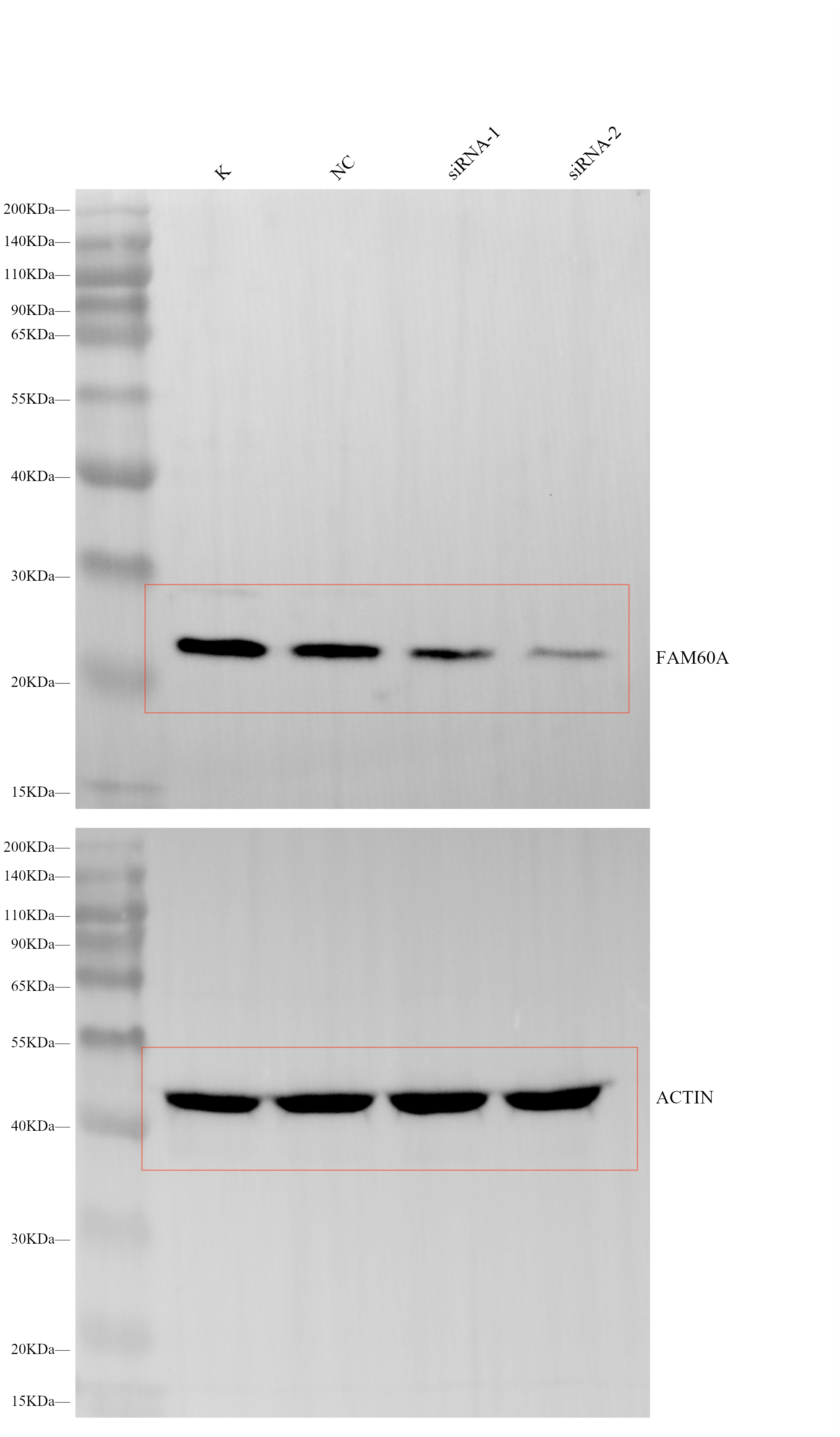

Supplement: Supplementary file 6 — Figure S6. [file CAM4-12-17491-s003.tif]

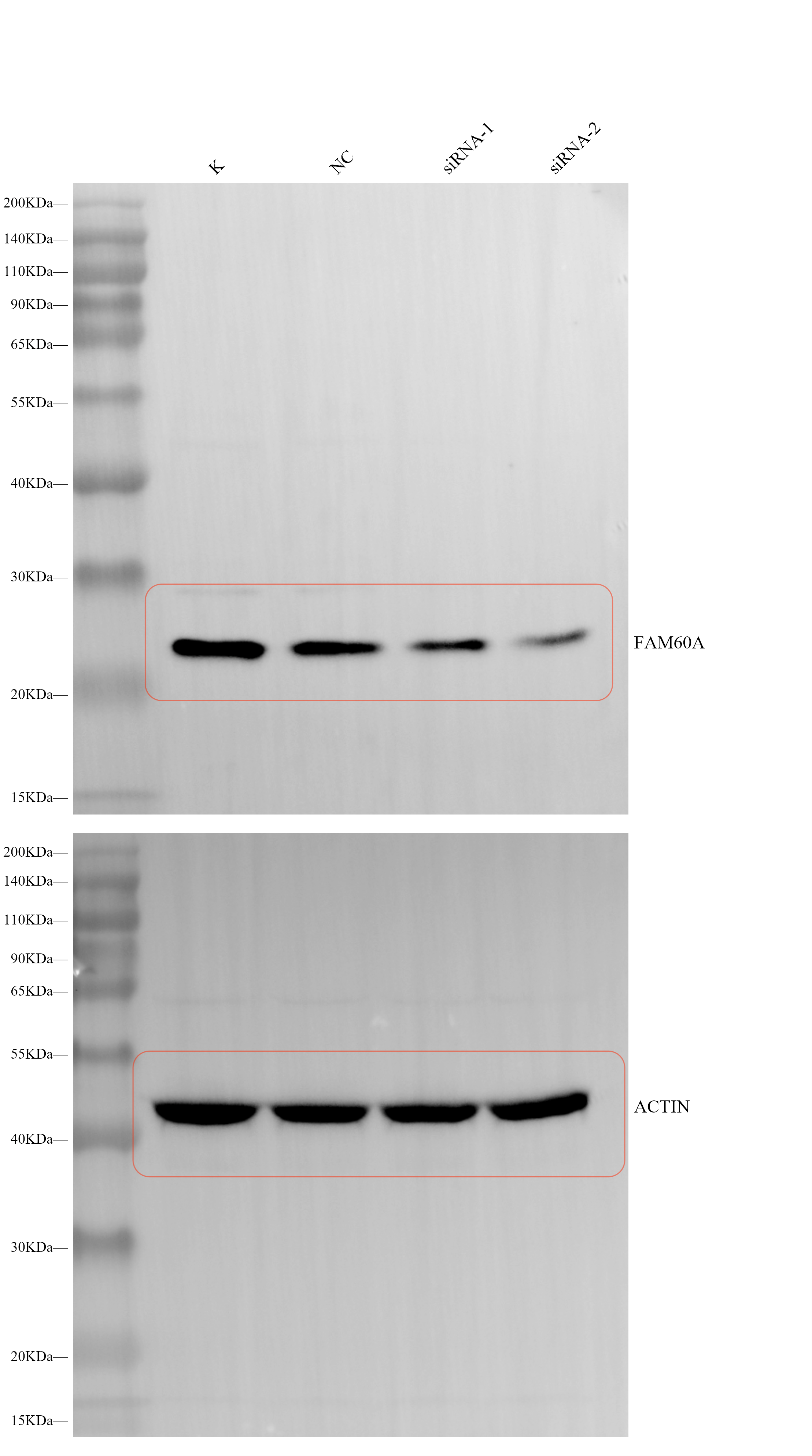

Supplement: Supplementary file 7 — Figure S7. [file CAM4-12-17491-s009.tif]

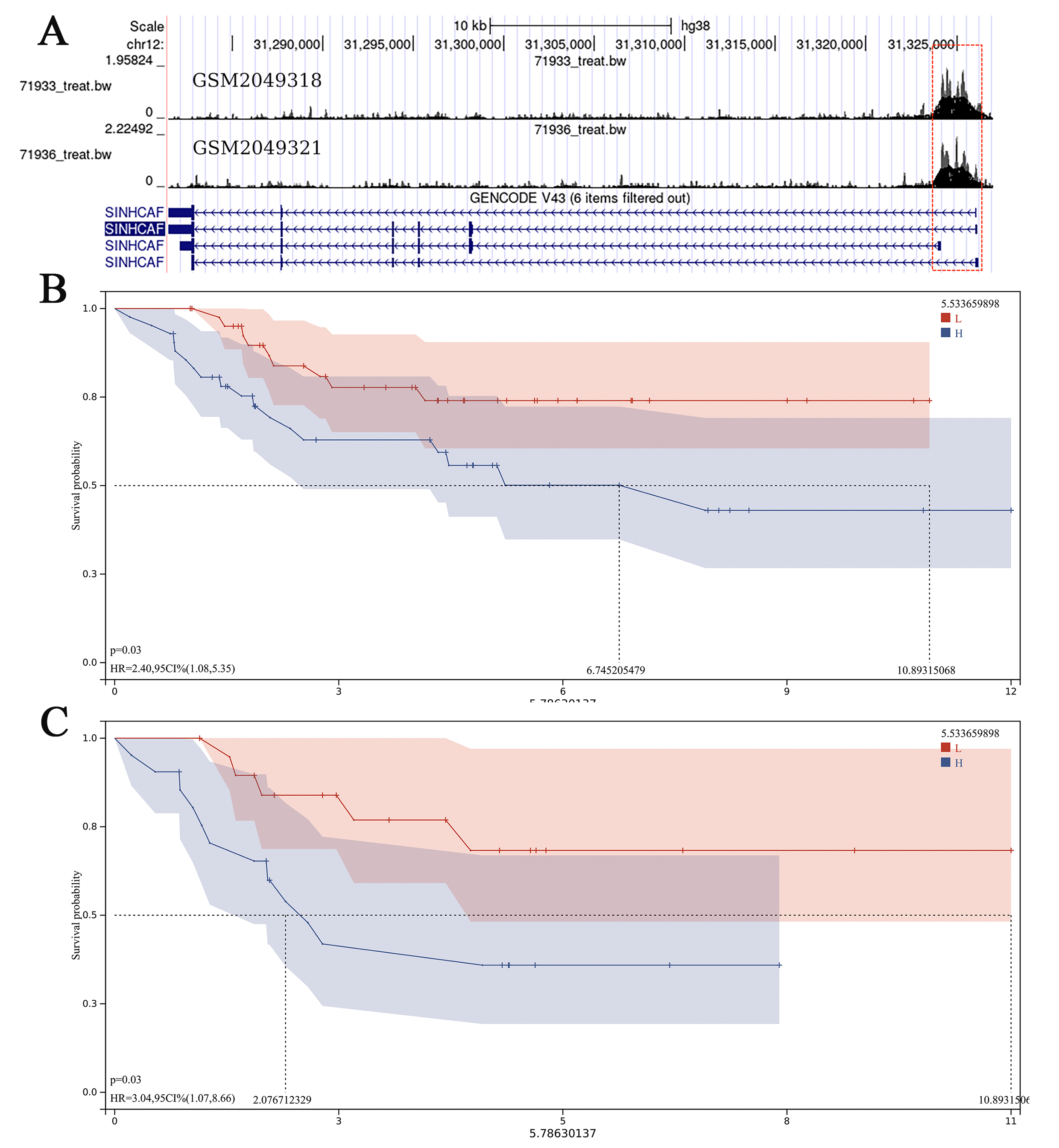

Supplement: Supplementary file 8 — Figure S8. [file CAM4-12-17491-s002.tif]

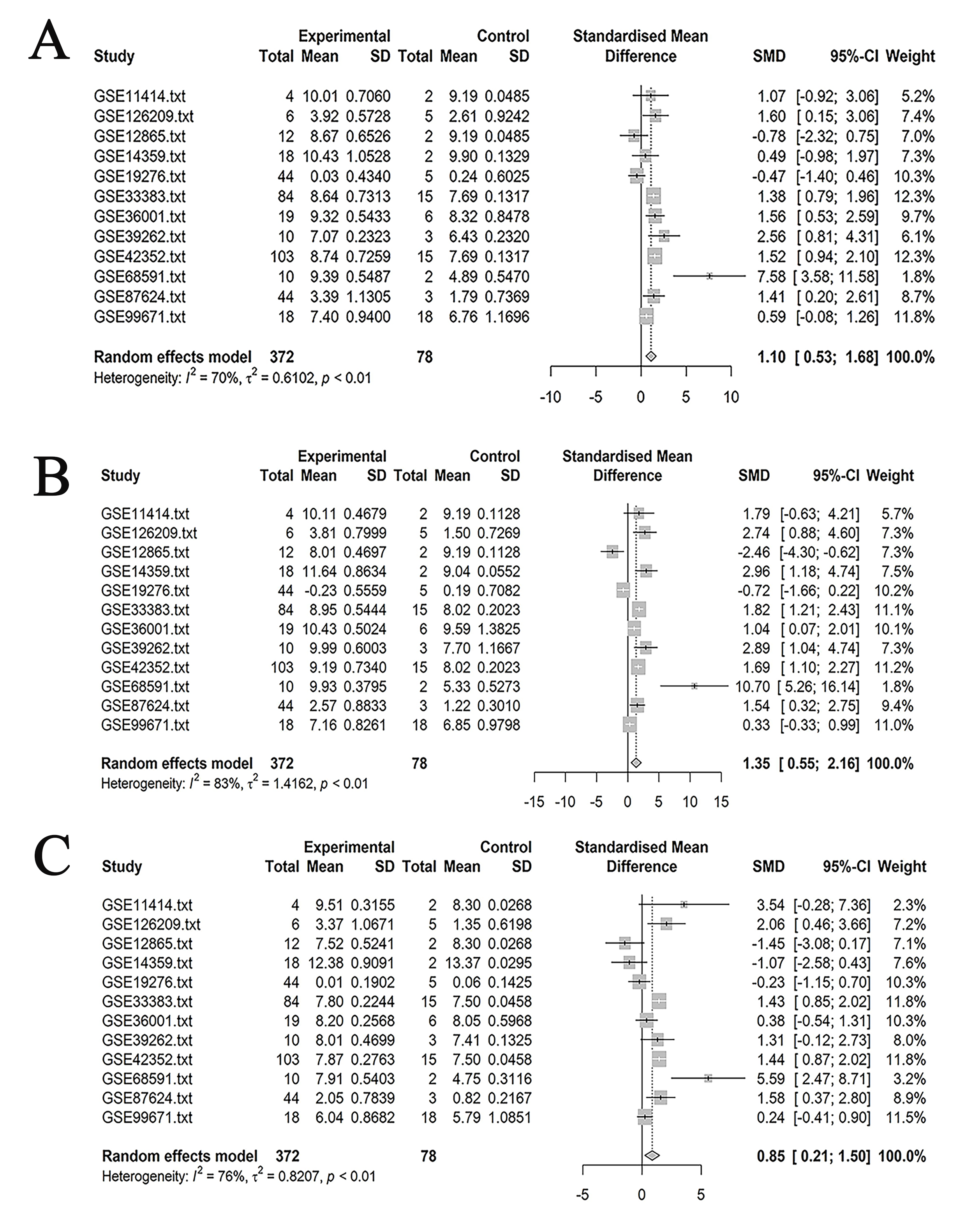

Supplement: Supplementary file 9 — Figure S9. [file CAM4-12-17491-s005.tif]
